# Coronary Artery Aneurysm: Its Evaluation in a 27-Year-Old Female Patient, Prognosis, and Suggested Treatment Strategies

**DOI:** 10.7759/cureus.47010

**Published:** 2023-10-14

**Authors:** Mohamad Zayour, Houssein Y Toufayli, Noura W Masri, Samer Terro, Elie Chammas

**Affiliations:** 1 Cardiology, University of Balamand, Beirut, LBN; 2 Cardiology, Faculty of Medicine, University of Balamand, Beirut, LBN; 3 Cardiology, Clemeceau Medical Center, Beirut, LBN

**Keywords:** coronary computed tomoangiography, echo cardiogram, adult congenital heart disease, left main coronary artery aneurysm, heart surgery

## Abstract

Coronary artery aneurysm (CAA) is characterized by a localized dilation of one or more of the coronary arteries with multiple etiologies, including congenital, acquired, or connected to auto-inflammatory diseases with multiple shapes and classifications. It is usually diagnosed incidentally during coronary imaging and can have variable clinical outcomes, ranging from asymptomatic to sudden cardiac death with a generally poor prognosis. Management of this condition faces a clinical dilemma due to the lack of clear guidelines or randomized trials. Treatment should be individualized based on symptoms, shape, and comorbidities. Herein, we present the case report of a 27-year-old female patient with no prior medical conditions. However, she presented with palpitations, and a compressive mass located over the right atrium was identified in the patient. After undergoing cardiac catheterization and coronary scanning, a giant aneurysm of the sinoatrial branch was detected with an aneurysmal left main that was retrieved surgically with good recovery and postoperative course.

## Introduction

Coronary artery aneurysm (CAA) is typically an incidental condition found during coronary imaging and can have a wide range of clinical manifestations, ranging from asymptomatic to acute coronary syndrome and sudden cardiac death [1,2]. The management of this condition is currently unclear due to the variety of presentations and the absence of clear recommendations [2,3]. The choice between monitoring, angioplasty, or surgical treatment should be discussed on a case-by-case basis [2].

## Case presentation

**Figure 1:**
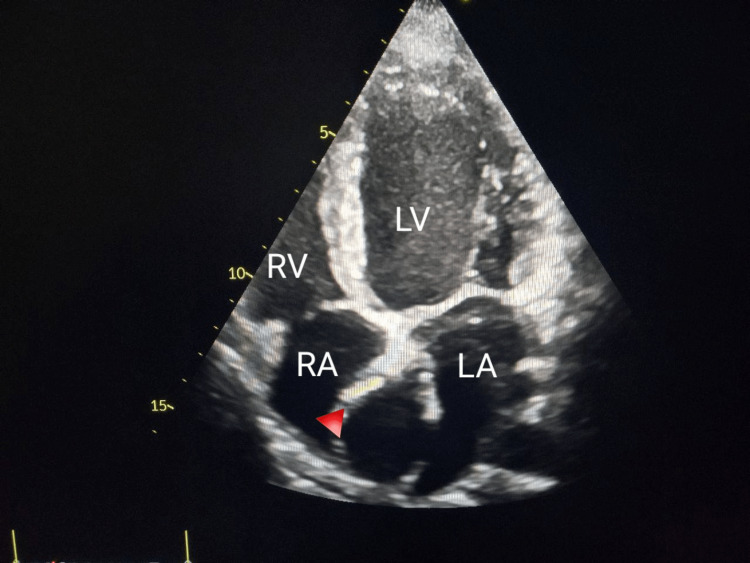
An echocardiogram apical four-chamber view showing mass compression in the right atrium (arrowhead). RA: right atrium; RV: right ventricle; LA: left atrium; LV: left ventricle

We present herein the case of a 27-year-old female with a previous clear medical history. However, she presented with palpitations and had undergone an echocardiography. The echocardiogram (Figure 1) showed good left and right ventricular systolic function with no major valvular heart disease.

**Figure 2:**
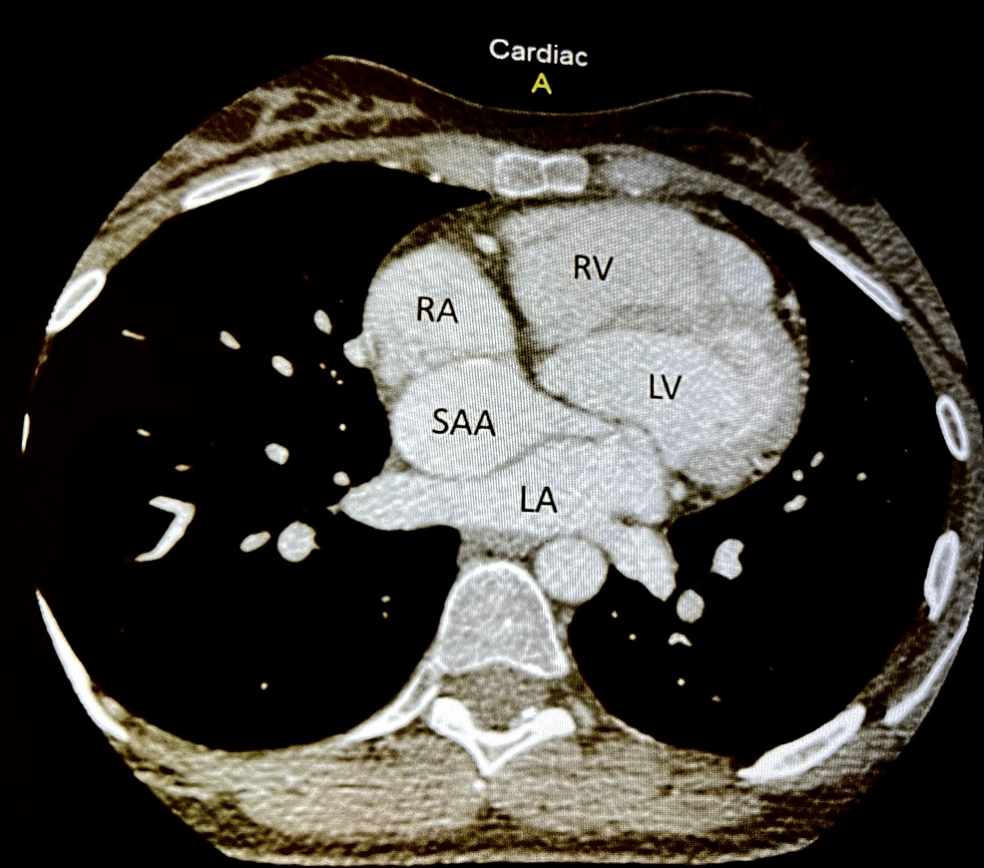
A coronary scan showing the sinoatrial branch aneurysm compressing the right atrium. SAA: sinoatrial aneurysm; RA: right atrium; RV: right ventricle; LA: left atrium; LV: left ventricle

**Figure 3:**
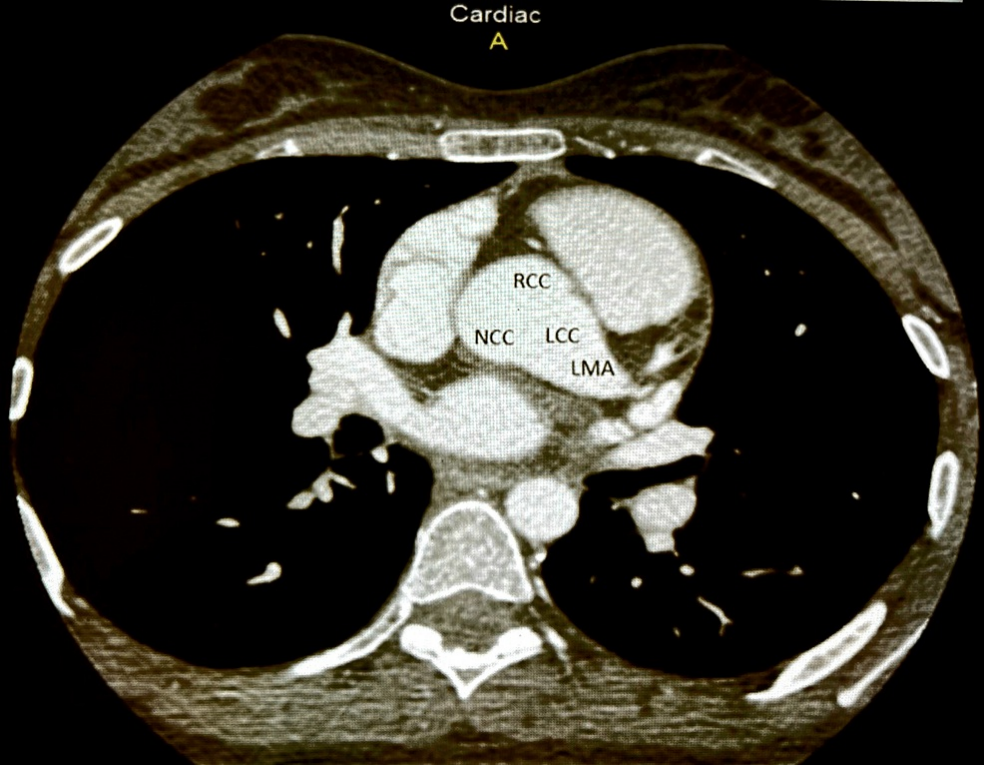
A coronary scan showing the left main aneurysm. LMA: left main aneurysm; RCC: right coronary cusp; LCC: left coronary cusp; NCC: non-coronary cusp

However, it revealed a large mass compressing the right atrium, which contained a blood flow that seemed diastolic and appeared to originate from the left coronary sinus. A subsequent coronary computerized tomography scan (CT-scan) (Figures 2-3) revealed a dilated left main artery measuring 11 mm that bifurcated into the left anterior descending artery (LAD) and the left circumflex artery (LCX).

**Figure 4:**
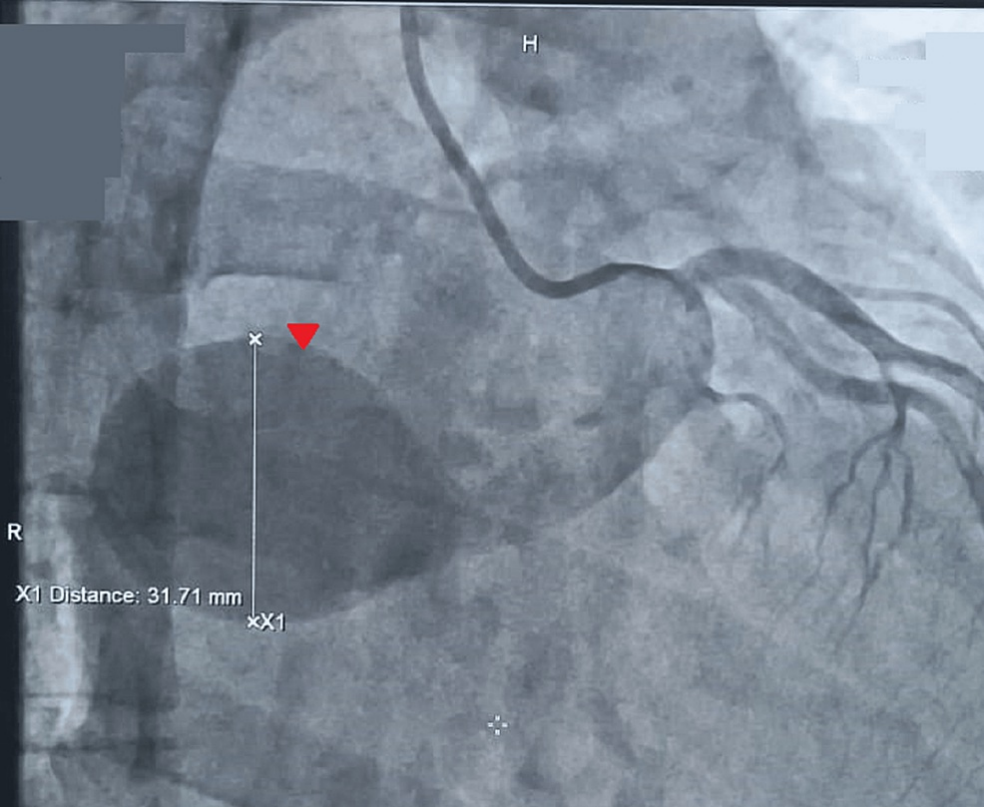
A coronary angiogram in the right anterior oblique cranial view showing an aneurysmal sinoatrial branch (arrowhead).

**Figure 5:**
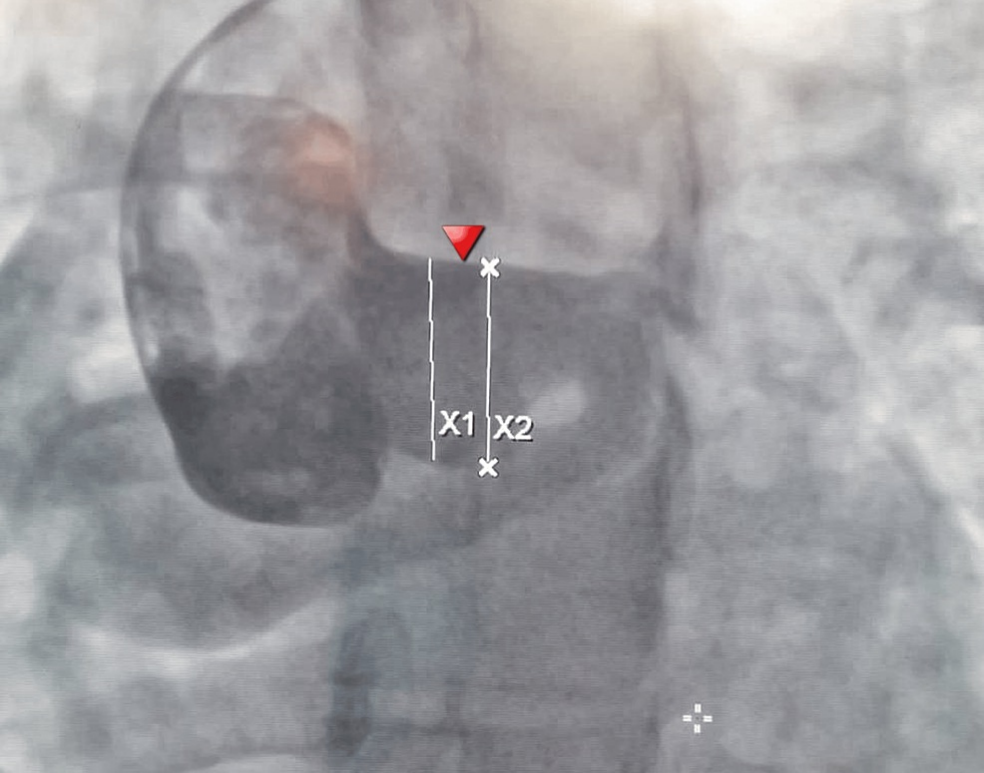
A coronary angiogram in the left anterior oblique view showed an aneurysmal left main coronary artery (arrowhead).

The LAD was free of disease, but the LCX had a large aneurysmal artery measuring 12 mm at the proximal part and 9 mm at the mid-part, with severe aneurysmal dilation measuring 33 mm x 37 mm distally. These findings are confirmed by coronary angiogram (Figures 4-5).

The patient was treated surgically with double ligation using clips at the level of the left main trunk, distally on the roof of the right atrium, as well as ligature used at the central part of the fistula. This resulted in the direct collapse of the aneurysm and the disappearance of the risk without any noted electrical or hemodynamic changes. The patient had a smooth post-operative course with no major complications.

## Discussion

CAA is characterized by the localized expansion of the coronary artery surpassing the 1.5-fold diameter of the adjacent healthy segment and classified according to shape, vessel wall composition, size, and extent of involvement.

 The frequency is around 5% accidentally found during cardiac catheterization in an old study [4]. It may be congenital, acquired, or a result of an inflammatory disease. The main cause behind this condition in adults is atherosclerosis, while, in children, it is primarily attributed to Kawasaki disease [1].

CAAs are classified as "giant" either when their diameter is four times more than the reference vessel diameter, or when they have a diameter larger than 8 mm [5]. It is an asymptomatic progressive condition that is incidentally discovered through coronary angiography; it can have a poor outcome, even in the absence of associated coronary artery disease. It is considered an independent predictor of mortality, with studies showing that up to 71% of patients with CAAs might survive for a period of five years [6]. It has the potential to cause life-threatening complications, including but not limited to rupture, compression of nearby cardiopulmonary structures, formation of blood clots with subsequent embolization to distal areas, as well as the development of a fistula or involvement of nearby cardiac structures [1,2,7].

If the transverse diameter is greater than the longitudinal diameter, CAAs are classified as saccular aneurysms; however, they are classified as fusiform aneurysms if the opposite is true [2].

According to the results of an angiographic study, men are more likely to have CAAs. The study revealed the presence of this disease in 1.79% (89 cases) of the 4.970 men compared to 0.56% (12 cases) of the 2,131 women who underwent the study [8].

It is common for patients with CAAs to exhibit symptoms such as angina pectoris, dyspnea, edema, or sudden death. However, it is challenging to determine whether these symptoms are specifically related to the CAAs, or if they are instead manifestations of other conditions that frequently accompany aneurysms, such as coronary atherosclerosis, coronary thrombosis, coronary stenosis, coronary vasculitis, acute myocardial infarctions, or old myocardial infarctions [8].

According to current research, the prevalence of authentic CAA is reported to be less than 1%. The right coronary artery is typically most affected, accounting for 40% of cases, followed by the left anterior descending artery at 32%. The left main artery is the least commonly affected, with a prevalence of 3.5% [2,9,10].

It is worth noting that saccular aneurysms were found to be more frequent in the left anterior descending coronary artery compared to other coronary arteries [2,4,11]. Atherosclerotic and vasculitis CAAs tend to involve multiple arteries, whereas congenital and iatrogenic CAAs typically affect only one vessel [2].

The treatment strategy for CAA is personalized and contingent upon various factors encompassing the aneurysm's attributes, such as shape, size, and location; technical complexities; and clinical circumstances depending on whether CAA is asymptomatic or may manifest as an acute coronary syndrome, leading to its detection, as well as any coexisting atherosclerosis [3].

Furthermore, there is a significant gap in our understanding of individuals with CAA, whether they are symptomatic or asymptomatic, due to the scarcity of randomized trials and limited availability of extensive data.

The pathogenesis of a significant number of CAA cases, especially in elderly patients, is associated with atherosclerosis. Therefore, it is crucial to prioritize intensive risk factor modification in these patients. The optimal management strategy for patients with CAA or coronary artery ectasia (CAE), particularly those incidentally diagnosed, remains a subject of ongoing discussion. Whether dual antiplatelet or therapeutic anticoagulation should be employed is still debated [2]. However, if there is concern regarding thrombosis and/or embolism, long-term usage of antiplatelet and anticoagulant therapy is preferable.

The potential connection between inflammatory cytokines and matrix metalloproteinases (MMPs) with CAAs may also suggest a possible role for statins and renin-angiotensin system inhibition. However, there is currently a lack of studies supporting these hypotheses.

In the case of Kawasaki disease, intravenous immunoglobulin (IVIG) therapy is employed for the treatment of CAAs in affected patients [4,6,12]. Nevertheless, it is important to take into account the concerns associated with the utilization of covered stents including decreased deliverability, potential risks of restenosis and thrombosis, as well as the possibility of obstructing side branches [12,13].

Coil insertion is an alternative percutaneous approach for treating wide-necked aneurysms; however, it requires expertise to carry out such interventions. It is important to note that coil herniation can potentially result in the occlusion of the main blood vessel making stent-assisted techniques the preferred choice. Additionally, there is a risk of aneurysm rupture during the manipulation of microcatheters, coils, or wires, which should be taken into consideration [12,14,15].

Surgery serves as an alternative for patients who are not suitable candidates for percutaneous treatment. It is also recommended for individuals with obstructive coronary artery disease or those with large saccular aneurysms at a high risk of rupture. Various procedures can be carried out during surgery, such as aneurysm resection, proximal and/or distal ligation, in addition to aneurysmal thrombectomy and aneurysmectomy with or without bypass grafting [12,16].

It is essential to thoroughly deliberate surgical and nonsurgical therapeutic choices with the patient. While many experts concur that the mere existence of CAAs does not necessitate operative intervention, the severity of accompanying coronary artery stenosis is the primary consideration when determining the need for surgical treatment in patients with CAAs. Surgical intervention may be appropriate for symptomatic patients who display indications of emboli from the aneurysm to the distal coronary bed, causing myocardial ischemia [17].

Here, in this case, despite the high operative risk, a decision was made to pursue surgical management for the symptomatic patient due to the combination of her young age, active lifestyle, and large aneurysmal size after a thorough discussion.

## Conclusions

In this case report, we describe the case of a 27-year-old female patient without any preexisting health issues. Subsequently, she sought medical attention due to palpitations. Further evaluation revealed the presence of a compressing mass over the right atrium. Following cardiac catheterization and coronary scanning, a giant aneurysm of the sinoatrial branch was identified, and the patient was treated surgically. Managing this condition poses a challenge due to the absence of clear guidelines or randomized trials. Therefore, treatment approaches should be personalized, considering factors such as symptoms, aneurysmal shape, and comorbidities. Overall, the management of CAAs requires a multidisciplinary approach involving interventional cardiologists, cardiothoracic surgeons, and other relevant specialties. Further research and evidence-based guidelines are needed to establish standardized protocols for the treatment and follow-up of patients with coronary aneurysms, taking into account individual patient characteristics and clinical presentations.
